# Beyond the scaffold: extracellular matrix uptake in breast cancer

**DOI:** 10.1042/BST20250121

**Published:** 2026-07-08

**Authors:** Elena Rainero

**Affiliations:** School of Biosciences, University of Sheffield, Western Bank, Sheffield S10 2TN, U.K.

**Keywords:** breast cancers, cancer cell metabolism, cancer-associated fibroblasts, extracellular matrix, tumour microenvironment

## Abstract

Breast cancer is associated with a highly fibrotic tumour microenvironment, where cancer-associated fibroblasts (CAFs) secrete excessive amounts of extracellular matrix (ECM). Fibrosis and collagen deposition correlate with poor patient survival, indicating that the ECM plays a role in promoting tumorigenesis. The ECM is constantly remodelled through extracellular and intracellular degradation pathways. While protease-dependent extracellular ECM degradation has been well studied, the intracellular degradation pathway is less understood. Here, I will describe the evidence supporting a role for ECM internalisation and lysosomal degradation in promoting breast cancer progression. Both cancer cells and CAFs are reported to uptake ECM components via different ECM receptors. These include TEM8 in fibroblasts, Endo180 in both cancer cells and CAFs, and α2β1 integrin in cancer cells. Importantly, this process has been associated with metabolic reprogramming under nutrient deprivation conditions representative of the breast cancer TME, cancer cell growth *in vitro* and *in vivo*, and cancer cell migration and invasion. Therefore, regulators of ECM endocytosis and lysosomal delivery might represent novel potential targets to prevent tumour growth and metastasis in ECM-rich breast cancers.

## Introduction

Breast cancer is the most commonly diagnosed cancer in the world [1], and the most aggressive subtype, triple-negative breast cancer, is associated with extensive fibrosis in the tumour microenvironment (TME). This is driven by the excessive deposition of extracellular matrix (ECM) components, primarily by cancer-associated fibroblasts (CAFs). The ECM is a complex 3D network of secreted proteins, playing important roles in both physiological and pathological conditions. Indeed, the ECM controls a variety of cell functions, including cell adhesion, polarity, proliferation, migration, metabolism, and differentiation, which play a role in tissue homeostasis and, when deregulated, are associated with cancer progression [2]. Highly fibrotic, or desmoplastic, tumours are associated with reduced survival in breast cancer patients [3,4]. Consistently, different ECM components, including collagens and hyaluronic acid, have been reported to accumulate in the TME, leading to a stiffer stroma, which promotes tumour growth and correlates with worse patient prognosis [5].

The ECM in the TME is constantly remodelled via cycles of synthesis, deposition, and degradation. Extensive literature has defined the role of secreted proteases in the cleavage and degradation of ECM components. These include matrix metalloproteases (MMPs), a disintegrin and metalloproteinase, a disintegrin and metalloproteinase with thrombospondin motifs, and cathepsins [6]. An emerging alternative ECM degradation pathway involves ECM component internalisation, followed by lysosomal degradation [7]. Here, I will describe the molecular mechanisms underpinning this process in both breast cancer and stromal cells, its implications in tumour progression, and the potential to exploit these pathways for anti-cancer interventions.

## The ECM as a nutrient source for cancer cells

The breast cancer TME is characterised by hypoxia and nutrient starvation [8]. While nutrient levels in the TME in breast cancer have not been systematically characterised, analysis of the metabolite content of pancreatic tumours indicates reduced levels of glucose and amino acids [9]. This pushes cancer cells to adopt nutrient scavenging pathways to support metabolism and energy production. Macropinocytosis, a non-specific endocytosis pathway controlling the engulfment of fluids and proteins, has emerged as a key survival mechanism in pancreatic cancer [10]. Indeed, macropinocytosis has been shown to sustain cell metabolism by promoting the uptake of plasma proteins (such as albumin), nucleotides, ECM components, lipids, and cellular debris [10]. Interestingly, nutrient deprivation has been shown to promote the expression of ECM-related genes in fibroblasts [11], suggesting that low nutrient levels in the TME might favour ECM accumulation.

**Figure 1:**
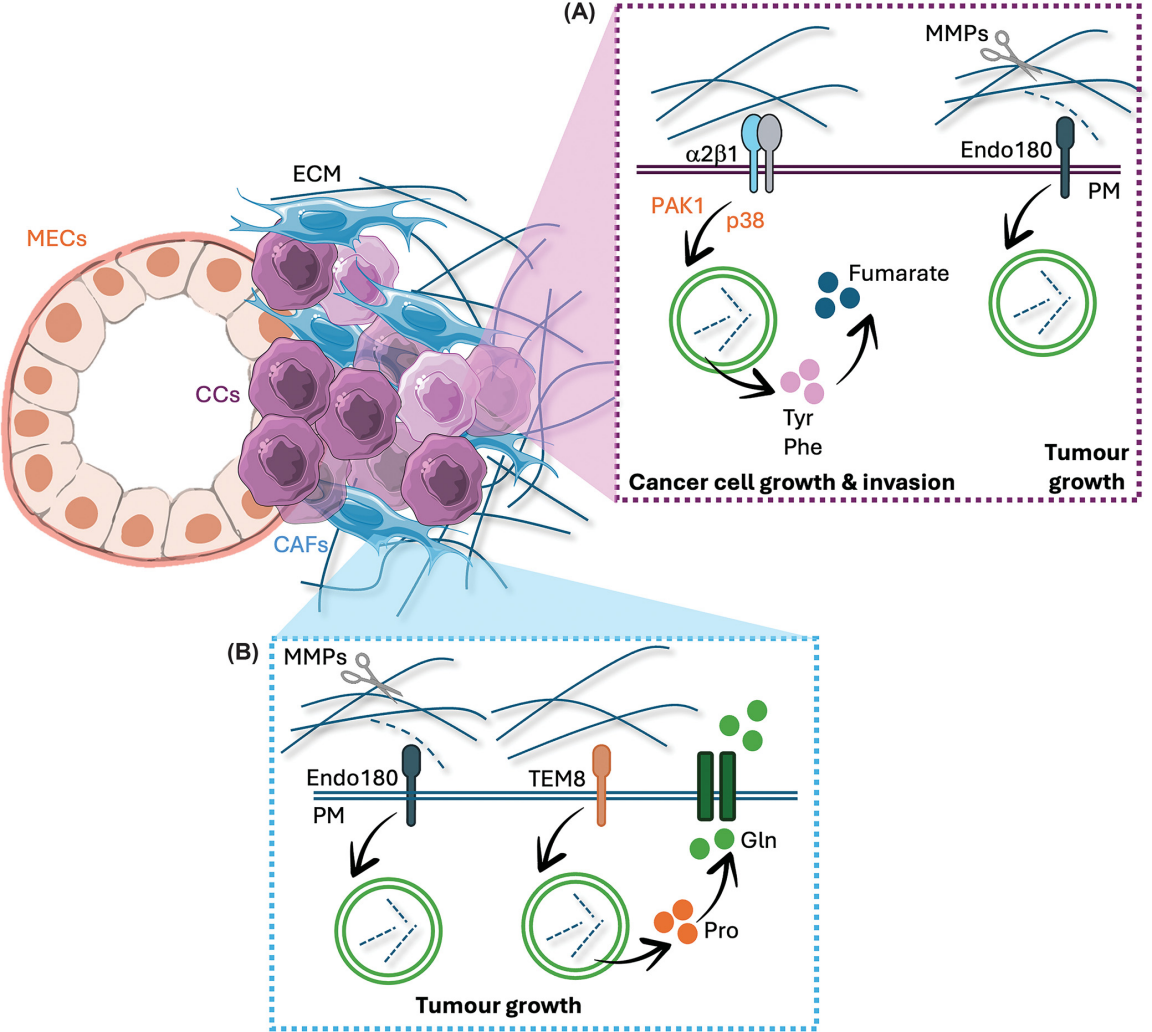
ECM internalisation in the breast cancer TME (**A**) In breast cancer cells, α2β1 integrin promotes PAK1-dependent ECM uptake by tuning the activation of p38/mitogen-activated protein kinase (MAPK). ECM lysosomal degradation results in intracellular accumulation of tyrosine and phenylalanine, which are catabolised to fumarate to support energy production. This pathway promotes cancer cell growth and invasion. MMP-generated collagen fragments are internalised in an Endo180-dependent manner and promote tumour growth. (**B**) In cancer-associated fibroblasts, Endo180- and TEM8-dependent collagen uptake drives tumour growth. Proline extraction from internalised collagen fibres is catabolised to glutamine, which supports the growth of both CAFs and cancer cells. MEC, mammary epithelial cells; CCs, cancer cells; CAFs, cancer-associated fibroblasts; ECM, extracellular matrix; MMPs, matrix metalloproteases; PAK1, p21-activated kinase 1; Tyr, tyrosine; Phe, phenylalanine; PM, plasma membrane; Pro, proline; Gln, glutamine. Schematics were obtained from bioart on a CC-BY licence (bioart.niaid.nih.gov/bioart/559; bioart.niaid.nih.gov/bioart/433; bioart.niaid.nih.gov/bioart/431).

Our recent work demonstrated that the ECM supported the growth of invasive breast cancer cells, but not non-transformed or non-invasive cells, under amino acid starvation. To determine the endocytic pathway(s) mediating ECM internalisation, breast cancer cells were seeded on fluorescently labelled laminin-rich basement membrane matrix, collagen I, or fibroblast-generated cell-derived matrices and treated with pharmacological inhibitors blocking the small GTPase dynamin (involved in clathrin- and caveolin-dependent endocytosis), lipid raft-mediated endocytosis, and macropinocytosis. We found that, across all ECM types, macropinocytosis inhibition strongly reduced ECM uptake. Consistent with the idea that the ECM represents a nutrient source to fuel cell growth under starvation, inhibition of ECM internalisation significantly prevented ECM-dependent cell growth. Mechanistically, ECM lysosomal degradation resulted in increased intracellular amino acid content, with tyrosine and phenylalanine being the most up-regulated. Tyrosine catabolism led to the production of fumarate and was required for ECM-dependent cell growth (Figure 1A), suggesting that amino acid catabolism might support TCA cycle progression under starvation [12].

Interestingly, ECM-specific endocytic responses could be observed, with macropinocytosis inhibition eliciting the strongest reduction in collagen I-rich matrix uptake, while dynamin and lipid raft inhibition reduced laminin-rich basement membrane matrix and cell-derived matrix uptake, without affecting collagen I uptake. Consistently, the down-regulation of dynamin 2/3 or caveolin 1/2 strongly reduced laminin-rich basement membrane matrix and cell-derived matrix uptake [12]. This indicates that different endocytic pathways are likely to control the internalisation of different ECM components, which warrants further investigation. Interestingly, caveolin-mediated endocytosis has been shown to contribute to nutrient scavenging in pancreatic cancer under nutrient-deprived conditions [13]. Indeed, albumin supplementation rescued the growth of PANC-1 and MIA PaCa-2 pancreatic cancer cells under glutamine starvation in control cells, but not in caveolin 1-deficient cells. Similarly to ECM internalisation, multiple pathways are involved in controlling albumin uptake. Macropinocytosis has been shown to be strongly activated by glutamine deprivation through the activation of epidermal growth factor signalling in AsPC-1 and HPAF-II pancreatic cancer cells, which are characterised by low basal macropinocytosis, while no change upon glutamine deprivation was observed in PANC-1 cells, which have a higher basal macropinocytic rate [14]. More recently, this pathway was shown to be controlled by atypical protein kinase C [15]. It is therefore possible that glutamine deprivation promotes albumin uptake either via caveolin-dependent endocytosis or macropinocytosis in a cell line-dependent manner.

Cells interact with the ECM through plasma membrane receptors, including integrins, dystroglycan, discoidin domain receptors, and urokinase plasminogen activator receptor protein (uPARAP/Endo180), member of the mannose receptor family. Integrins are heterodimers composed of an α and a β subunit. 18 α and 8 β subunits give rise to 24 heterodimers. In agreement with early studies in fibroblasts [16], we demonstrated that α2β1 integrin was required for ECM internalisation [17]. Indeed, pharmacological inhibition and siRNA-mediated protein down-regulation resulted in a significant reduction in the ability of breast, pancreatic, and ovarian cancer cells to uptake collagen I and fibroblast-generated cell-derived matrices (Figure 1A). Mechanistically, this was mediated by the fine-tuning of p38 mitogen-activated protein kinase, as both the activating kinase (MAP3K1) and the inhibitory phosphatase (PP2A) were required for efficient ECM endocytosis [17]. Internalised ECM was co-trafficked with α2β1 through early and late endosomes/lysosomes, eventually resulting in protein degradation [17]. Invasive breast cancer cells were shown to internalise ECM while migrating through fibrillar matrices and inhibition of ECM uptake impaired cancer cell migration and invasion [17].

While primarily expressed in stromal cells (see below), Endo180 has also been shown to control ECM trafficking in epithelial cancer cells. Indeed, Endo180 was reported to be up-regulated in a subset of basal-like breast cancer cells. This was mediated by transforming growth factor β stimulation and copy number gains and amplification. The overexpression of WT Endo180, but not an internalisation-deficient mutant, resulted in increased tumour growth in xenograft experiments (Figure 1A) [18], suggesting that Endo180-mediated collagen uptake supported tumour growth. It is unclear whether the removal of collagen from the stroma or its role in nutrient scavenging is important in this context. Similarly, Endo180 promoted collagen uptake and cell invasion in pancreatic cancer [19] and glioblastoma cells [20], suggesting that Endo180 contribution to tumour progression is not limited to breast cancer but could be a more general feature shared among different cancer types.

Together, evidence so far supports an important role for ECM uptake in promoting cancer cell growth by supporting energy production via nutrient scavenging. While ECM endocytosis also contributes to cell migration and invasion, it is less clear whether this is also linked with ATP production downstream of ECM lysosomal degradation. Further work is needed to elucidate this.

## CAFs internalise collagens to promote tumour growth

ECM internalisation has mostly been studied in the context of fibrosis, whereby intracellular collagen degradation by fibroblasts controls matrix turnover in an Endo180-dependent manner [21]. Indeed, Endo180 was shown to be required for the internalisation and lysosomal delivery of collagen IV and collagen V [22]. Similarly, in the polyoma middle T (PyMT) murine breast cancer model, Endo180 was reported to be strongly expressed in mesenchymal cells embedded in the stromal compartment around tumours. Importantly, collagen IV internalisation was observed in control explants from PyMT mice, while in Endo180-deficient tumours collagen IV remained mostly pericellular. Consistently, intracellular collagen accumulation was observed in Endo180-positive tumours *in vivo* by transmission electron microscopy [23]. Finally, Endo180 genetic ablation prevented breast tumour growth (Figure 1B), suggesting that stromal collagen internalisation has a pro-tumorigenic role [23]. As only fluorescently labelled collagen IV was used in these experiments, it is not clear whether this pathway is also responsible for the internalisation of other collagen types, such as collagen I, which is highly abundant in breast tumours.

In the E0771 orthotopic murine breast cancer model, collagen I was shown to be predominantly internalised by stromal components, including macrophages and CAFs, in an MMP-dependent manner [24]. It is important to note that collagen internalisation experiments here were performed by incubating single cell suspensions with soluble collagen I to mimic the endocytosis of cleaved ECM fragments. This may not reproduce physiological ECM uptake in cells embedded in fibrillar collagen I in a complex TME. Furthermore, the molecular mechanisms controlling collagen I endocytosis were not explored.

In addition to Endo180, collagen I uptake has been shown to be modulated by tumour endothelial marker 8 (TEM8) in the tumour stroma in different murine cancer models (including breast cancer), with CAFs being the most abundant population in desmoplastic tumours [25]. TEM8 was shown to bind to collagen I and VI, but not collagen IV, and to be required for collagen I internalisation and lysosomal degradation. Interestingly, while stromal TEM8 was able to support tumour growth *in vivo*, the knock-in of a TEM8 mutant unable to bind and internalise collagen I was unable to do so, indicating that stromal collagen uptake supported cancer cell growth. Moreover, TEM8 expression was shown to be increased by growth factor and glutamine deprivation, suggesting that this pathway might be promoted in a nutrient-starved TME [25]. It would be interesting to determine the molecular mechanisms through which nutrient limitation promotes TEM8 expression in stromal cells. As described above, collagens have been identified as an unconventional nutrient source in the TME. Proline represents ∼23% of collagen and can be metabolised to generate ATP. In stromal cells, proline can be converted to glutamine, which can potentially represent an energy source for starved cancer cells. Indeed, Hsu et al. demonstrated that proline derived from internalised collagen I is converted into glutamine, which supports the survival of stromal cells but can also be secreted into the TME to be taken up by cancer cells [25] (Figure 1B). It is important to note that these experiments were performed in co-cultures comprising stromal cells and colon, lung, or pancreatic cancer cells, in which collagen I does not support cell growth under starvation. It would be interesting to test the contribution of CAF-derived glutamine in the context of breast or pancreatic cancer cells, which are also able to internalise and degrade ECM. Nevertheless, these observations support the idea of a pan-cancer contribution of stromal ECM uptake to tumour progression, which is not limited to breast cancer.

CAFs are a heterogenous population, with different subtypes identified in the TME. The most abundant are myofibroblasts (myCAFs), inflammatory (iCAFs), and antigen-presenting [26]. While systematic studies directly addressing the ability of the different CAF subtypes to internalise ECM components are lacking, FAP^+^ myCAFs have been identified as the main TME component mediating collagen I uptake in an E0771 breast cancer model [24]. Consistently, TEM8 expression was used to separate myCAFs from iCAFs in breast cancer [27]. In PDAC, macropinocytosis has been shown to be required for the maintenance of the myCAF phenotype under glutamine starvation [28]; however, it is unclear whether ECM uptake specifically plays a role in this context. Together, these observations suggest that myCAF might be the predominant CAF subtype mediating ECM uptake.

## Matrix scavenging as a therapeutic target

**Figure 2:**
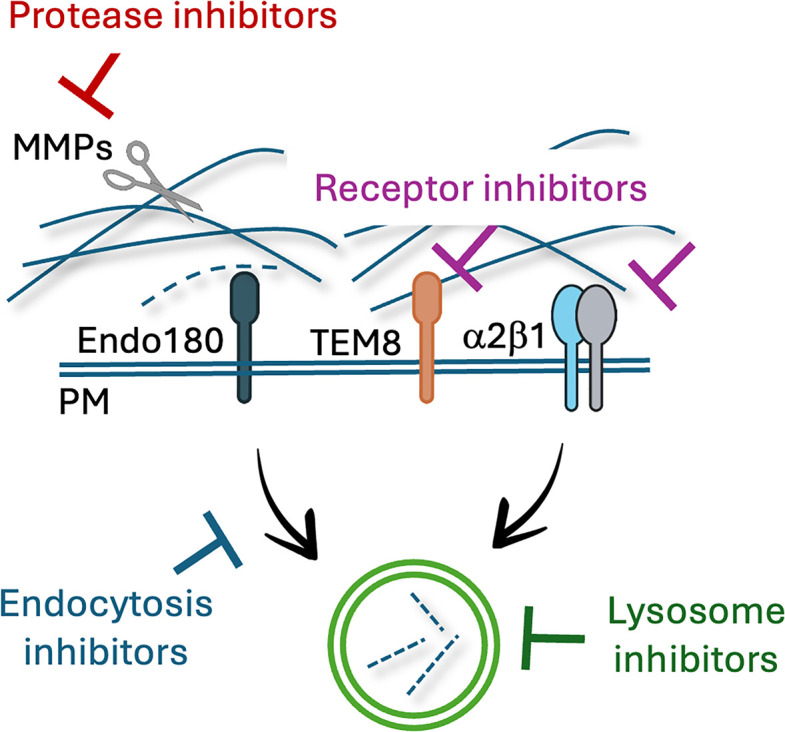
Strategies to target ECM uptake Several stages can be targeted by small molecule inhibitors. These include protease inhibitors to prevent Endo180-dependent collagen uptake. Alternatively, inhibitors can interfere with receptor/collagen binding. Endocytosis inhibitors block the internalisation steps, while lysosomal inhibitors prevent ECM degradation. PM, plasma membrane; MMPs, matrix metalloproteases.

Given the body of evidence suggesting that matrix scavenging supports tumour progression, pharmacological interventions blocking this process hold promise as new anti-cancer agents. Different strategies could be used to prevent ECM uptake and degradation, including MMP, ECM receptor, endocytosis, and lysosomal inhibitors (Figure 2).

MMP inhibitors were developed over three decades ago, but early clinical trials for different cancer types were not successful, mostly due to toxicity and lack of understanding of MMP biology. More specific MMP inhibitors were recently developed, and clinical trials indicate that they do not elicit the off-target effects observed in the earlier generation of inhibitors. The broad-spectrum MMP inhibitor Periostat has been approved by the Food and Drug Administration for the treatment of periodontal disease [29], indicating that it could be repurposed as an anti-cancer agent. Endo180 is internalised via clathrin-mediated endocytosis. Since native collagen fibres are too large for clathrin-coated vesicles, MMP-mediated collagen cleavage is required for Endo180-mediated uptake [21]. However, the internalisation of collagen fibrils, independent of Endo180 and mediated by α2β1 integrin, does not require MMP-dependent proteolysis. Indeed, we did not detect any change in fibrillar collagen I internalisation and collagen I-dependent breast cancer cell growth in the presence of the broad-spectrum MMP inhibitor GM6001 [12]. Similarly, collagen I internalisation in lung cancer cells was reported to be MMP-independent [30]. Therefore, MMP inhibition could be a promising strategy in contexts in which ECM uptake is primarily modulated by Endo180.

While there are no Endo180 pharmacological inhibitors, an inhibitor of α2 integrin expression (E7820) has been tested in Phase I clinical trials in patients with advanced malignancy. The drug was well-tolerated, but the best response observed was stable disease [31,32]. An ongoing trial is assessing its efficacy in unresectable solid tumours in Japan [33]. No clinical trials focusing on breast cancer have been found. However, E7820 lacks specificity, as it was also reported to inhibit β1, α3, and α5 integrin [34], as well as promote the degradation of the splicing factor RBM39 [33]. The small molecule inhibitor BTT-3033, which prevents α2β1 collagen binding, has been used in pre-clinical studies and has been reported to be well tolerated in mice [35]. Similarly, a TEM8-blocking antibody has been shown to reduce tumour growth in mouse models [25]. It would be interesting to see whether these inhibitors could be moved forward to the clinic in the context of highly fibrotic tumours.

Endo180 is internalised in a clathrin-dependent manner, while collagen-bound α2β1 is mostly endocytosed via macropinocytosis [12,17]. Therefore, targeting these trafficking pathways might be a potential strategy to prevent ECM uptake and ECM-driven cancer progression. Inhibitors blocking clathrin-mediated endocytosis were tested in clinical trials to inhibit infection by SARS-CoV-2, while DYN101, an antisense nucleotide targeting dynamin 2, was trialled for the treatment of centronuclear myopathy [36]. Although these trials did not deliver the expected outcomes, the blockers tested showed a good tolerance, suggesting that they might be repurposed to prevent nutrient scavenging. While several small molecules used to inhibit macropinocytosis have pleiotropic effects, more recent studies identified novel compounds that could be clinically relevant. These include imipramine, a tricyclic antidepressant used to treat anxiety and depression [37], and virapinib, a small molecule shown to prevent macropinocytosis-dependent viral entry [38].

Finally, lysosomal function is required for intracellular ECM degradation, linked to the pro-tumorigenic functions described above. Therefore, lysosomal inhibitors hold promise to prevent ECM uptake-dependent tumorigenesis. Chloroquine and hydroxychloroquine are FDA-approved agents that block lysosomal acidification [39]. The autophagy inhibitor Lys05 has been reported to accumulate within and deacidify lysosomes, therefore preventing lysosomal hydrolysis. Interestingly, significant single-agent anti-tumour activity has been observed, with no toxicity in mice treated with lower doses of Lys05 [40].

It is important to note that the inhibition of ECM internalisation might result in the exacerbation of fibrosis in the TME, as collagen might accumulate when the intracellular degradation pathway is blocked. Therefore, it would be interesting to assess the effect of these approaches in combination with anti-fibrotic drugs.

## Conclusions

While the contribution of protease-dependent extracellular ECM degradation is well-established and linked with cancer cell invasion and metastasis, much less is known about the intracellular degradation pathway, mediated by ECM internalisation followed by lysosomal degradation. Different receptors and endocytic pathways have been shown to contribute to ECM uptake, both in cancer cells and in stromal cells.

Compelling evidence supports a role for ECM internalisation in promoting metabolic reprogramming, cancer cell growth, and invasion *in vitro* as well as tumour progression *in vivo*. It remains to be established whether this is a druggable pathway in breast cancer. It would be interesting to determine the impact of ECM uptake on amoeboid versus mesenchymal cell migration, as these have different requirements for cell/ECM adhesion. More work is required to better understand the molecular machinery controlling ECM uptake and intracellular trafficking, as well as the mechanistic contribution of nutrient deprivation. This might lead to the identification of novel molecular players, which could be potential therapeutic targets. Since ECM-rich breast tumours correlate with poor prognosis and have been linked with tumour recurrence and metastasis [41], it is extremely timely and important to develop better strategies to prevent ECM-driven tumorigenesis.

## Perspectives

- The breast cancer tumour microenvironment is highly fibrotic, and the excessive accumulation of ECM promotes tumour progression and correlates with poor patient prognosis. Understanding how ECM promotes tumorigenesis will pave the way to the development of novel therapeutic strategies.
- Both cancer and stromal cells are able to internalise ECM components and degrade them in their lysosomes. This process is linked with cancer cell growth and migration, as well as tumour formation *in vivo*.
- Small molecules targeting ECM internalisation and intracellular trafficking might represent novel therapeutic options for fibrotic breast tumours.

## Open Access

Open access for this article was enabled by the participation of University of Sheffield in an all-inclusive Read & Publish agreement with Portland Press and the Biochemical Society under an agreement with JISC.

## Abbreviations

CAFs: cancer-associated fibroblasts
ECM: extracellular matrix
iCAFs: inflammatory CAFs
MAPK: mitogen-activated protein kinase
MMPs: matrix metalloproteases
myCAFs: myofibroblasts CAFs
PyMT: polyoma middle T
TEM8: tumour endothelial marker 8
TME: tumour microenvironment
uPARAP/Endo180: urokinase plasminogen activator receptor protein
